# *Astragaloside IV* combined with *ligustrazine* ameliorates abnormal mitochondrial dynamics via Drp1 SUMO/deSUMOylation in cerebral ischemia–reperfusion injury

**DOI:** 10.1111/cns.14725

**Published:** 2024-04-14

**Authors:** Xiangyu Chen, Tong Yang, Yue Zhou, Zhigang Mei, Wenli Zhang

**Affiliations:** ^1^ Key Laboratory of Hunan Province for Integrated Traditional Chinese and Western Medicine on Prevention and Treatment of Cardio‐Cerebral Diseases College of Integrated Traditional Chinese and Western Medicine Hunan University of Chinese Medicine Changsha Hunan China; ^2^ The First Clinical Medicine School of Guangdong Pharmaceutical University Guangzhou Guangdong China; ^3^ Hunan Provincial Hospital of Integrated Traditional Chinese and Western Medicine Changsha Hunan China; ^4^ Third‐Grade Pharmacological Laboratory on Chinese Medicine Approved by State Administration of Traditional Chinese Medicine College of Medicine and Health Sciences China Three Gorges University Yichang Hubei China; ^5^ School of Pharmacy Hunan University of Chinese Medicine Changsha Hunan China

**Keywords:** *Astragaloside IV*, cerebral ischemia–reperfusion injury, *Ligustrazine*, mitochondrial dynamics, SUMOylation

## Abstract

**Objectives:**

*Astragaloside IV* (AST IV) and *ligustrazine* (Lig), the main ingredients of *Astragali Radix* and *Chuanxiong Rhizoma* respectively, have demonstrated significant benefits in treatment of cerebral ischemia ‐reperfusion injury (CIRI); however, the mechanisms underlying its benificial effects remain unclear. SUMO‐1ylation and deSUMO‐2/3ylation of dynamin‐related protein 1 (Drp1) results in mitochondrial homeostasis imbalance following CIRI, which subsequently aggravates cell damage. This study investigates the mechanisms by which AST IV combined with Lig protects against CIRI, focusing on the involvement of SUMOylation in mitochondrial dynamics.

**Methods:**

Rats were administrated AST IV and Lig for 7 days, and middle cerebral artery occlusion was established to mimic CIRI. Neural function, cerebral infarction volume, cerebral blood flow, cognitive function, cortical pathological lesions, and mitochondrial morphology were measured. SH‐SY5Y cells were subjected to oxygen–glucose deprivation/reoxygenation (OGD/R) injury. Mitochondrial membrane potential and lactic dehydrogenase (LDH), reactive oxygen species (ROS), and adenosine triphosphate (ATP) levels were assessed with commercial kits. Moreover, co‐immunoprecipitation (Co‐IP) was used to detect the binding of SUMO1 and SUMO2/3 to Drp1. The protein expressions of Drp1, Fis1, MFF, OPA1, Mfn1, Mfn2, SUMO1, SUMO2/3, SENP1, SENP2, SENP3, SENP5, and SENP6 were measured using western blot.

**Results:**

In rats with CIRI, AST IV and Lig improved neurological and cognitive functions, restored CBF, reduced brain infarct volume, and alleviated cortical neuron and mitochondrial damage. Moreover, in SH‐SY5Y cells, the combination of AST IV and Lig enhanced cellular viability, decreased release of LDH and ROS, increased ATP content, and improved mitochondrial membrane potential. Furthermore, AST IV combined with Lig reduced the binding of Drp1 with SUMO1, increased the binding of Drp1 with SUMO2/3, suppressed the expressions of Drp1, Fis1, MFF, and SENP3, and increased the expressions of OPA1, Mfn1, Mfn2, SENP1, SENP2, and SENP5. SUMO1 overexpression promoted mitochondrial fission and inhibited mitochondrial fusion, whereas SUMO2/3 overexpression suppressed mitochondrial fission. AST IV combined with Lig could reverse the effects of SUMO1 overexpression while enhancing those of SUMO2/3 overexpression.

**Conclusions:**

This study posits that the combination of AST IV and Lig has the potential to reduce the SUMO‐1ylation of Drp1, augment the SUMO‐2/3ylation of Drp1, and thereby exert a protective effect against CIRI.

## Graphical abstract

*Astragaloside IV* and *Ligustrazine*, the main ingredients of medicinal and food homologous plants *Astragali Radix* and *Chuanxiong Rhizoma* could ameliorate abnormal mitochondrial dynamics via the Drp1 SUMO/deSUMOylation following cerebral ischemia–reperfusion injury both in vivo and in vitro.

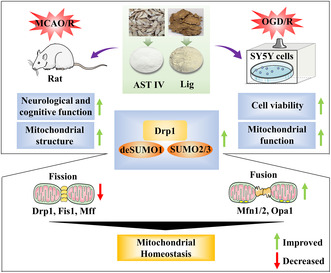

## 1 INTRODUCTION

Ischemic stroke, accounting for approximately 80% of all strokes, is characterized by a high rate of morbidity, disability, mortality, and recurrence, leading to significant suffering and financial burdens for families and society.^1^, ^2^ The primary objectives of clinical treatment for ischemic stroke are restoring blood flow as soon as possible, reviving the blood supply in the ischemic penumbra, and delaying brain tissue injury.^3^ Although blood flow recanalization can save dying neurons, ischemic stroke has a narrow window of treatment, and it can result in severe cerebral ischemia–reperfusion injury (CIRI), further worsening the patient's condition.^4^ Hence, preventing and treating CIRI have become the critical focus of clinical interventions for ischemic stroke.

Mitochondria are the primary target area for neuronal death after ischemia. The disruption of mitochondrial homeostasis appears to be a critical factor associated with CIRI.^5^ Regulating mitochondrial homeostasis relies on mitochondrial quality control (MQC), including mitochondrial biosynthesis, mitochondrial dynamics (mitochondrial fission/fusion), mitophagy,^6^, ^7^ and mitochondria‐derived vesicles.^8^ Recent studies demonstrated that dynamin‐related protein 1 (Drp1), mitochondrial fission protein 1 (Fis1), and mitochondrial fission factor (MFF) mediate mitochondria fission, while optic atrophy 1 (OPA1) and mitofusion1/2 (Mfn1/2) participate in mitochondrial fusion.^9^ CIRI could activate Drp1 and accelerate p62‐mediated autophagosome formation, leading to an inflammatory cascade, ROS overproduction, and autophagosome accumulation, aggravating the damage to mitochondria.^10^ In CIRI, mitochondrial dynamics are disrupted by excessive mitochondrial fission and insufficient mitochondrial fusion. Most strategies for mitochondrial dynamics focus on inhibiting mitochondrial fission or promoting mitochondrial fusion.^11^ Therefore, regulating mitochondrial dynamics is essential for maintaining mitochondrial homeostasis to mitigate CIRI and promote neurological function recovery in patients with ischemic stroke.

SUMOylation is a common form of protein posttranslational modification that involves a reversible, multistep enzymatic process similar to ubiquitination. The process results in adding a small ubiquitin‐like modifier (SUMO) to the target protein.^12^, ^13^, ^14^ Sentrin‐specific proteases (SENPs) can dissociate SUMO from the substrate protein in a process known as deSUMOylation.^15^ Drp1 is the target protein of SUMOylation, and its activity is regulated by SUMO1^16^ and SUMO2/3.^17^ SUMO‐1ylation and SENP3‐mediated deSUMO‐2/3ylation promote the mitochondrial localization of Drp1, leading to mitochondrial fragmentation.^18^, ^19^ SENP1, SENP2, and SENP5 facilitate Drp1 deSUMOylation, resulting in decreased Drp1‐SUMO1 levels, reduced protein stability, and ultimately reduced mitochondrial fragmentation.^20^, ^21^ The processes of SUMO/deSUMOylation of Drp1 play a key role in regulating mitochondrial dynamics.^22^ The intervention of Drp1 SUMOylation to control mitochondrial dynamics is expected to become a new target for anti‐CIRI.

*Astragali Radix* (Huangqi)^23^ and *Chuanxiong Rhizoma* (Chuanxiong)^24^ are medicinal and food homologous plants in traditional Chinese medicine, which rhizomes are the main edible medicinal parts. Consuming the rhizome with food is a typical therapy to keep healthy. *Astragali Radix*‐*Chuanxiong Rhizoma* is a widely used medicinal combination in Chinese herbal medicine for the treatment of ischemic stroke.^25^ However, the mechanisms underlying its therapeutic effects remain unclear. Hence, the primary active ingredients of these two herbs are combined to investigate potential mechanisms. *Astragaloside IV* (AST IV) is the active ingredient extracted from *Astragali Radix*, and *ligustrazine* (Lig) is an alkaloid extracted from *Chuanxiong Rhizoma*. Previous studies revealed that both AST IV and Lig play neuroprotective roles through anti‐inflammatory, antioxidative, and antiapoptosis pathways.^26^, ^27^ AST IV could attenuate mitochondrial damage by downregulating Drp1 expression, reduce mitochondria‐dependent apoptosis,^28^ promote the continuous production of SUMO1, and enhance angiogenesis under hypoxic conditions.^29^ Lig could prevent mitochondrial fragmentation by restoring the alterations in Drp1 and Mfn2 expressions^30^ and target mitochondrial transcription factor A (TFAM) and Drp1 and endoplasmic reticulum stress to alleviate mitochondrial abnormality and induce neuroprotection.^31^ Moreover, AST IV combined with Lig exerts a good synergistic protective effect in CIRI.^32^ Combining AST IV and Lig has the potential to improve CIRI by repairing the neurovascular unit,^33^ inhibiting calcium influx, increasing cellular viability,^34^ and providing anti‐inflammatory, antioxidative, and antiapoptotic effects^35^, ^36^; however, the mechanism of this effect remains unknown.

In this study, we hypothesized that combining AST IV and Lig might regulate Drp1 SUMO/deSUMOylation, thereby improving mitochondrial dynamics and ultimately ameliorating CIRI. An in vivo rat CIRI model and an in vitro SH‐SY5Y cell model of oxygen–glucose deprivation/reoxygenation (OGD/R) were established to explore the potential protective effects of combining AST IV with Lig against CIRI; and further to elucidate the potential mechanism of the active ingredients against CIRI by Drp1 SUMO/deSUMOylation and mitochondrial dynamics.

## 2 MATERIALS AND METHODS

### 2.1 Experimental animals

In total, 100 SPF male Sprague–Dawley rats (weight 250–280 g) were purchased from the Hunan Slike Jingda Laboratory Animal Co., Ltd. (license No. SCXK, Xiang, 2019‐0004). All rats were housed in the Hunan University of Traditional Chinese Medicine Laboratory Animal Center (license No. SCXK, Xiang, 2019‐0009) under temperatures of 20–25°C, humidity of 50%–70%, 12‐h light/dark cycle, and good ventilation with free access to water and food. The rats were allowed to adapt to the new environment for 7 days before the experiment. All experimental protocols and animal‐related activities adhered to the National Institute of Health Guide for the Care and Use of Laboratory Animals. Furthermore, the experimental protocol was approved by the Laboratory Animal Ethical Committee of Hunan University of Chinese Medicine (No. LL2021030201).

### 2.2 Drug preparation and treatment schedule

AST IV (C_41_H_68_O_14_, molecular weight = 784.97, purity >98%) was purchased from Shanghai Yuanye Bio‐Technology Co., Ltd., China, while Lig (C_8_H_12_N_2_, molecular weight = 136.19, purity >98%) was obtained from Sigma‐Aldrich Co., LLC., USA. AST IV and Lig powders were dissolved in saline with dimethyl sulfoxide (DMSO) to obtain the final doses of 40 and 80 mg kg^−1^, respectively. Then, the rats were randomly divided into five groups: sham group, I/R group, AST IV group, Lig group, and AST IV + Lig group. The rats in the AST IV and Lig groups were orally gavaged with the corresponding drug solutions once a day for 7 days before surgery. The AST IV + Lig group was given both AST IV (40 mg kg^−1^) and Lig (80 mg kg^−1^) by gavage. Concurrently, the sham and I/R groups received the same amount of normal saline.

### 2.3 Establishment of the CIRI model

The CIRI model was established by middle cerebral artery occlusion (MCAO), as previously described.^37^ Rats were anesthetized with an intraperitoneal injection of 2% pentobarbital sodium (50 mg kg^−1^) and fixed on a surgical manipulation plate in a supine position for skin preparation and disinfection. Then, a small incision was made along the midline of the neck, and the muscle tissue was gently separated. The proximal end of the common carotid artery (CCA) and the distal end of the external carotid artery (ECA) were ligated. A suitable monofilament nylon suture (Beijing Cinontech Co. Ltd., China) was carefully inserted into the internal carotid artery (ICA) through a small hole opened on the CCA to occlude the blood flow. Moreover, the monofilament nylon suture was gradually removed after 2 h of occlusion to allow for a 24 h reperfusion of blood flow. In the sham group, rats were not implanted in the monofilament nylon suture, but the same surgical procedures were performed as in the model group. Throughout the procedure, the rats were placed on an electric blanket to keep their body temperature stable.

### 2.4 Evaluation of neurological deficit

The neurological functions of all rats were evaluated using Zea‐Longa scores^38^ according to the following standards: 0, no neurological dysfunction (normal walk); 1, mild neurological dysfunction (incomplete extension of the paralyzed side forepaw); 2, moderate neurological dysfunction (turn toward the paralyzed side during walking); 3, severe neurological dysfunction (lean toward the paralyzed side during walking); 4, the most severe neurological dysfunction (inability to walk automatically and loss of consciousness). The rats with neurological function scores of 0–3 were included in the experiment as 0 score was obtained in the sham group.

### 2.5 Evaluation of cerebral infarct volume

The cerebral infarct volume was visualized using 2,3,5‐triphenyl‐tetrazolium chloride (TTC, Sigma‐Aldrich Co. LLC., USA) staining. The brain sample was harvested 24 h after reperfusion and placed at −20°C for 30 min. Then, the brain was evenly sliced into five pieces along the coronal plane. These sections were placed in a six‐well plate containing a 2% solution of TTC at 37°C for 30 min and fixed in a 4% polyformaldehyde solution for 24 h. Subsequently, pictures were taken, and the cerebral infarction volume was calculated using Image‐Pro Plus 6.0. The percentage infarct volume was calculated as follows: (infarct area/area of the whole section) × 100%.

### 2.6 Observation of cerebral blood flow

Laser speckle imaging was used to observe cerebral blood flow (CBF) in rats. The rat was placed on the brain stereotactic device. A cranial window measuring 4 × 6 mm^2^ was polished on the right side of the skull while keeping the dura mater intact. Subsequently, the Moor FLPI‐2 (Moor Instruments Ltd., Axminster, UK) blood flow imager was employed to observe CBF before MCAO, after MCAO, and after 24 h reperfusion.

### 2.7 Morris water maze test

The Morris water maze was used to test the spatial memory and learning abilities of the rats.^39^ During 7‐day training, the rats were placed in the pool from four quadrants with the aim of finding an invisible platform once a day for 60 s, respectively. If the rat failed to locate the platform in 60 s, it would be guided toward the platform and remain on the platform for 20 s. On the eighth day, the platform was removed to commence the space exploration experiment. Then, each rat was given 60 s to search for the platform. The escape latency, number of platform crossings, swimming time, and distance in the target quadrants were recorded and analyzed as measures of performance. If the rat failed to locate the station in 60 s, the track was terminated, and a score of 60 s was assigned.

### 2.8 Hematoxylin–eosin (HE) and Nissl staining

The rats were anesthetized and perfused transcardially with normal saline, followed by 4% paraformaldehyde. The brain samples were removed and fixed in 4% paraformaldehyde for 24 h. Then, each sample was embedded in paraffin and sectioned at 4 μm coronally. The brain slices were subjected to HE and Nissl staining according to standard protocols. Histopathological changes were subsequently observed under a light microscope. Images of three randomly selected high‐power fields were evaluated, and the number of Nissl‐positive cells was counted using Image‐Pro Plus 6.0.

### 2.9 Transmission electron microscope (TEM) evaluation

The brain tissue of each group was preserved in electron microscopy stationary liquid overnight at 4°C to evaluate mitochondrial morphology. Subsequently, the tissue was washed, fixed, dehydrated, infiltrated, embedded, and sectioned. Then, 70 nm thick ultrathin sections were stained with 2% uranyl acetate‐saturated alcohol and 2% lead citrate. The mitochondria morphology was observed under a TEM, and images were collected for analysis.

### 2.10 Cell culture, oxygen–glucose deprivation, and reoxygenation

The SH‐SY5Y cells were cultured in Dulbecco's modified Eagle medium (DMEM) containing 10% fetal bovine serum (FBS) and 1% antibiotics at 37°C under a humidified atmosphere of 5% CO_2_ and 95% O_2_. The cells were randomly assigned to different experimental groups. The cells at 40% density were seeded into six‐well plates and cultured for 12 h for subsequent experiments. The OGD/R model was established as a model of CIRI in vitro. Then, the complete culture medium was removed. The SY5Y cells were cultured with glucose‐free DMEM and incubated for 4 h at 37°C in a humidified atmosphere of 94% N_2_, 5% CO_2_, and 1% O_2_. Following the OGD treatment, the cells were cultured with a complete culture medium under normoxic conditions.

### 2.11 Experimental grouping

In the first set of experiments, the cells were divided into three groups: normal group, OGD/R group, and AST IV + Lig group. In the second set of experiments, the cells were divided into six groups: OGD/R group, AST IV + Lig group, OV‐SUMO1 group, OV‐SUMO1 + AST IV + Lig group, OV‐SUMO2/3 group, and OV‐SUMO2/3 + AST IV + Lig group. The cells in the normal group were cultured normally, while the remaining groups were established as OGD/R models.

### 2.12 Assessment of cell viability

The Cell Counting Kit‐8 (CCK‐8) assay was used to evaluate the cell viability of SY5Y cells and screen suitable concentrations and treatment times of AST IV and Lig for subsequent experiments. The cells were cultivated in 96‐well microplates at 1 × 10^5^ cells per well for 12 h. AST IV (final concentrations: 0, 3.125, 6.25, 12.5, 25, and 50 μM) and Lig (final concentrations: 0, 40, 80, 120, 140, and 160 μM) were taken 100 μL and added to the 96‐well microplates for 24 h to obtain a final concentration. Then, 10 μL of CCK8 was added to each well and incubated for 3 h at 37°C. The absorbance was measured at 450 nm. Moreover, 100 μL of AST IV and Lig were added to OGD/R cells and cultured for 0, 12, 24, 36, 48, and 72 h to observe the effect of AST IV combined with Lig on OGD/R cell proliferation. CCK8 was used to measure cell viability.

### 2.13 Cell transfection

SUMO1 and SUMO2/3 plasmids were designed and synthesized to achieve SUMO1 and SUMO2/3 gene overexpression. OV‐SUMO1 sequences were as follows: F‐5′ACGCGTCGACATGTCTGACCAGGAGGCAAAACCTT3′; R‐5′CCGCTCGAGCTAAACTGTTGAATGACCCCCCGTT3′. OV‐SUMO2/3 sequences were as follows: F‐5′CCGGAATTCATGGCCGACGAAAAGCCCAA3′; R‐5′CCGCTCGAGTCAGTAGACACCTCCCGTCTGCTGT3′. A serum‐free medium, Opti‐MEM, was used to dilute the SUMO1 plasmid, SUMO2/3 plasmid, and Lipo2000. Lipo2000 was added to the diluted SUMO1 plasmid and SUMO2/3 plasmid, respectively. The mixed plasmid‐liposome solution was added to six‐well plates, supplemented with serum‐free medium to 2 mL, and cocultured with SY5Y cells for 8 h. Finally, the supernatant was removed and replaced with a normal culture medium for further culture for 16 h.

### 2.14 Detection of mitochondrial membrane potential

Mitochondrial Membrane Potential Assay Kit with JC‐1 (Beijing Solarbio Science & Technology Co., Ltd., China) was used to detect mitochondrial membrane potential. The JC‐1 dye (200×) was diluted 200 times. The cells cultured in six‐well plates were stained at 37°C for 20 min with 1 mL JC‐1 dye added to each well. The cells were detected by flow cytometry after washing, centrifugation, and suspension. The excitation wavelength was set at 490 nm, and the emission wavelength was set at 530 nm to detect JC‐1 monomers. For detecting JC‐1 aggregates, the excitation wavelength was set at 525 nm, and the emission wavelength was set at 590 nm.

### 2.15 Detection of ROS accumulation

A 2′,7′‐dichlorofluorescein diacetate (DCFH‐DA) fluorescent probe from a ROS Fluorometric Assay Kit (Elabscience Biotechnology Co., Ltd., China) was used to detect ROS generation. DCFH‐DA is a fluorescent probe without fluorescence that can freely cross the cell membrane. After entering cells, it can be hydrolyzed to DCFH by intracellular esterase. In the presence of ROS, DCFH is oxidized to DCF, a strong green fluorescent substance that cannot penetrate cell membranes. SY5Y cells were collected and incubated in DCFH‐DA and kept at 37°C for 30 min. The fluorescence intensity was measured using a fluorescent enzyme‐labeled instrument at excitation and emission wavelengths of 500 and 525 nm, respectively. The fluorescence intensity reflected ROS production.

### 2.16 Detection of ATPase activity

ATPase is an important high‐energy compound in living organisms that can maintain cell membrane potential and ion balance. ATPase catalyzes the ATP‐to‐ADP and inorganic phosphorus hydrolysis. The content of inorganic phosphorus can reflect ATPase activity. The SY5Y cells were homogenized and centrifuged. Subsequently, the supernatant was taken, and the ATPase activity was detected using the ATPase Activity Assay Kit (Elabscience Biotechnology Co., Ltd., China). The absorbance was measured at 640 nm with a microplate reader.

### 2.17 Detection of lactate dehydrogenase (LDH) release

LDH, a stable cytoplasmic enzyme ubiquitously found in most cells, exhibits rapid release into the cell culture supernatant upon plasma membrane injury. In this study, SY5Y cells were homogenized and centrifuged. The supernatant was extracted, and the LDH release was detected using the LDH Activity Assay Kit (Elabscience Biotechnology Co., Ltd., China) according to the manufacturer's instructions. Finally, the absorbance was monitored on a microplate reader at 450 nm.

### 2.18 Detection of Drp1 SUMOylation

Co‐IP was used to detect Drp1 SUMOylation. Then, brain cortex tissues and SY5Y cells were homogenized in IP lysis buffer and centrifuged. After adding the Drp1 antibody and normal rabbit IgG, the supernatant was incubated overnight at 4°C and then added to the pretreated protein A/G agarose beads. After Co‐IP, the supernatant was collected. The agarose beads were washed, collected, and mixed in IP lysis buffer and loading buffer. Western blotting was used to detect the expressions of SUMO1 (in vivo: 1:1000, ABclonal, China; in vitro: 1:2000, Proteintech, China) and SUMO2/3 (in vivo: 1:1000, ABclonal, China; in vitro: 1:500, Proteintech, China).

### 2.19 Western blot analysis

BCA Protein Colorimetric Assay Kit (Elabscience Biotechnology Co., Ltd., China) was used to quantify the total protein of brain cortex tissues and SY5Y cells. Samples with an equal amount of protein were loaded and separated via sodium dodecyl sulfate‐polyacrylamide gel electrophoresis (SDS‐PAGE). The proteins were transferred to the appropriate polyvinylidene fluoride membrane (Millipore, USA), blocked with 5% skimmed milk for 1 h, and thens incubated with diluted antibodies at 4°C overnight. For the in vivo experiments, Drp1 (1:2000), Fis1 (1:1500), MFF (1:8000), OPA1 (1:2000), Mfn1 (1:1000), Mfn2 (1:2000), and β‐actin (1:5000) antibodies were purchased from Proteintech Group, Inc., China. Meanwhile, for the in vitro experiments, the antibodies included Drp1 (1:800; AiFang Biological, China), Fis1 (1:1500; Bioss, China), OPA1 (1:1000; AiFang Biological, China), Mfn1 (1:1000; Affinity, China), Mfn2 (1:500; AiFang Biological, China), and SENP6 (1:1000; Abcam, USA). Furthermore, MFF (1:2000), SUMO1 (1:2000), SUMO2/3 (1:500), SENP1 (1:2000), SENP2 (1:1000), SENP3 (1:800), SENP5 (1:3000), and β‐actin (1:5000) were obtained from Proteintech Group, Inc., China. Then, the membranes were washed and incubated with secondary antibodies for 2 h at room temperature. After rewashing with TBST buffer, the membranes were added ECL reagent to enable the detection of the protein bands.

### 2.20 Statistical analysis

The experimental data were analyzed using SPSS 25 statistical software (IBM Corp., USA), and statistical graphs were plotted using GraphPad Prism 9.0 (GraphPad Software, Inc.). The data were expressed as means ± standard error of the mean (SEM). Shapiro–Wilk test was used for normality test. Multiple comparisons were performed using one‐way analysis of variance (ANOVA). If the assumptions of homogeneity of variance and normal distribution were met, the LSD test was used. If the normal distribution assumption was satisfied but not the homogeneity of variance assumption, Dunnett's T3 test was employed. Otherwise, the data were analyzed by the nonparametric Kruskal–Wallis tests. A value of *p* < 0.05 was considered statistically significant.

## 3 RESULTS

### 3.1 AST IV and Lig decelerated CIRI

**FIGURE 1:**
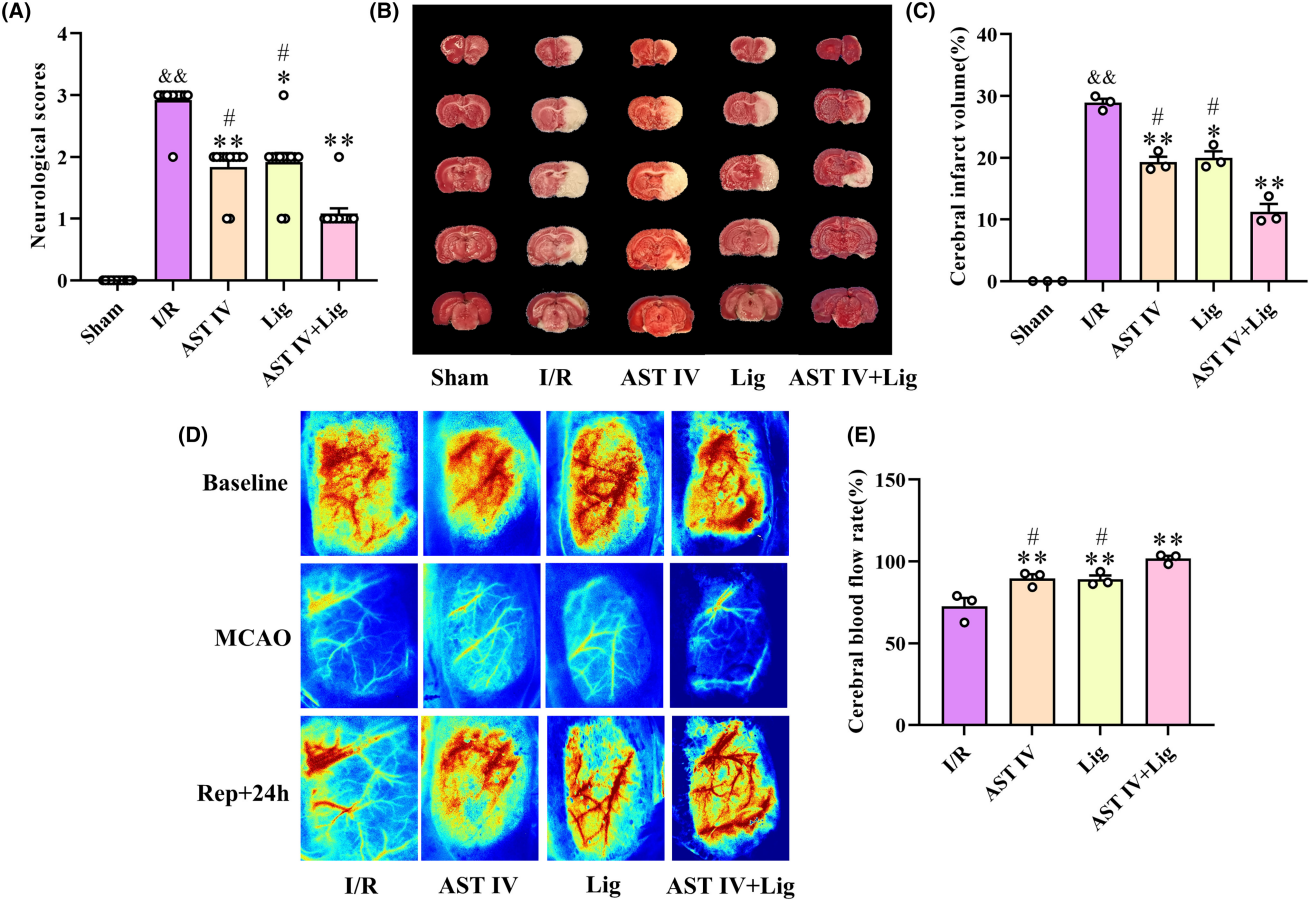
AST IV, Lig, and AST IV + Lig decelerated CIRI. (A) Neurological scores in rats after 24 h of reperfusion; (B) TTC staining of the infarct regions after CIRI; (C) Percentage of infarct volume; (D) Representative images of CBF; (E) Perfusion analysis of CBF after CIRI. ^&&^ *p* < 0.01, versus sham group; **p < 0.01, versus I/R group; *p < 0.05, versus I/R group; ^*#*^ *p* < 0.05, versus AST IV + Lig group, *n* = 3.

Neurological function scoring was used to observe the behavioral changes after 24‐h reperfusion (Figure 1A). No neurological defects were detected in the sham group. Conversely, the neurological function scores of rats in the I/R group were significantly higher than those in the sham group (*p* < 0.01). Compared with the I/R group, neurological function scores of rats in AST IV, Lig, and AST IV + Lig groups were significantly decreased (*p* < 0.01, *p* < 0.05, and *p* < 0.01, respectively). The improvement in neurological function in the AST IV + Lig group was significantly better than that in AST IV and Lig groups (both *p* < 0.05). The volume of cerebral infarction was observed using TTC staining (Figure 1B,C). The rats in the sham group had no cerebral infarction. Compared with the sham group, the volume of cerebral infarction in the I/R group was significantly increased (*p* < 0.01). In contrast, compared with the I/R group, the volume of cerebral infarction in the AST IV, Lig, and AST IV + Lig groups was significantly reduced (*p* < 0.01, *p* < 0.05, and *p* < 0.01, respectively). The cerebral infarct volume in the AST IV + Lig group was markedly lower than that in the AST IV and Lig groups (both *p* < 0.05). CBF in the right brain was monitored using laser speckle imaging to observe the recovery of CBF during the CIRI process in rats (Figure 1D,E). CBF was abundant in the right cerebral cortex before cerebral ischemia; however, after modeling, CBF decreased rapidly in each group. Compared with the I/R group, the cerebral blood reperfusion levels of rats in the AST IV, Lig, and AST IV + Lig groups were significantly increased (all *p* < 0.01). The cerebral blood reperfusion level in the AST IV + Lig group was significantly higher than that in AST IV and Lig groups (both *p* < 0.05).

### 3.2 AST IV and Lig ameliorated CIRI‐induced cognitive impairment

**FIGURE 2:**
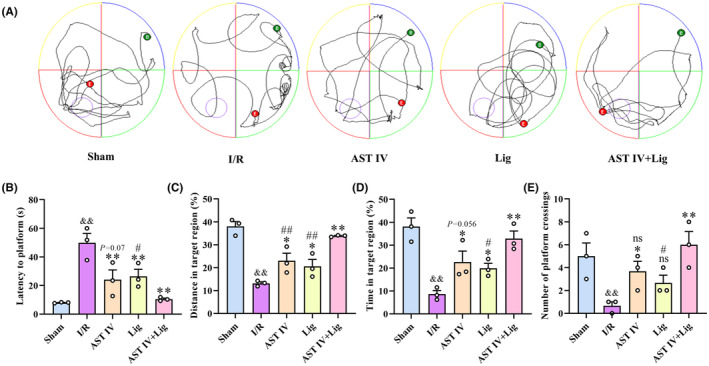
AST IV, Lig, and AST IV + Lig ameliorated CIRI‐induced cognitive impairment.(A) Swimming routes of rats; (B) Latency to platform; (C) Distance in target region; (D) Time in target region; (E) Number of platform crossings. ^&&^ *p* < 0.01, versus sham group; **p < 0.01, versus I/R group; *p < 0.05, versus I/R group; ^*##*^ *p* < 0.01, versus AST IV + Lig group; ^*#*^ *p* < 0.05, versus AST IV + Lig group, *n* = 3.

The cognitive function in rats was evaluated by the Morris water maze (MWM) test (Figure 2). Compared with the sham group, the latency to the platform of rats in the I/R group was significantly increased (*p* < 0.01), whereas the distance and time to the target quadrant and times of crossing the platform were reduced considerably (both *p* < 0.01). Compared with the I/R group, the latency to the platform of rats in AST IV and Lig groups was significantly decreased (both *p* < 0.01), whereas the distance and time to the target quadrant were significantly increased (*p* < 0.05). The number of platform crossings in the AST IV group was substantially higher than that in the I/R group (*p* < 0.05). Compared with the I/R group, the latency to the platform in the AST IV + Lig group was extremely significantly decreased (*p* < 0.01), whereas the distance and time to the target quadrant and the times of crossing the platform were extremely significantly increased (all *p* < 0.01). Moreover, the latency to the platform in the AST IV + Lig group was considerably shorter than that in the Lig group (*p* < 0.01). The distance to the target quadrant in the AST IV + Lig group was significantly higher than that in the AST IV and Lig groups (both *p* < 0.01). The time to the target quadrant and number of platform crossings in the AST IV + Lig group were significantly higher than those in the Lig group (*p* < 0.05).

### 3.3 AST IV and Lig alleviated pathological damage of CIRI

**FIGURE 3:**
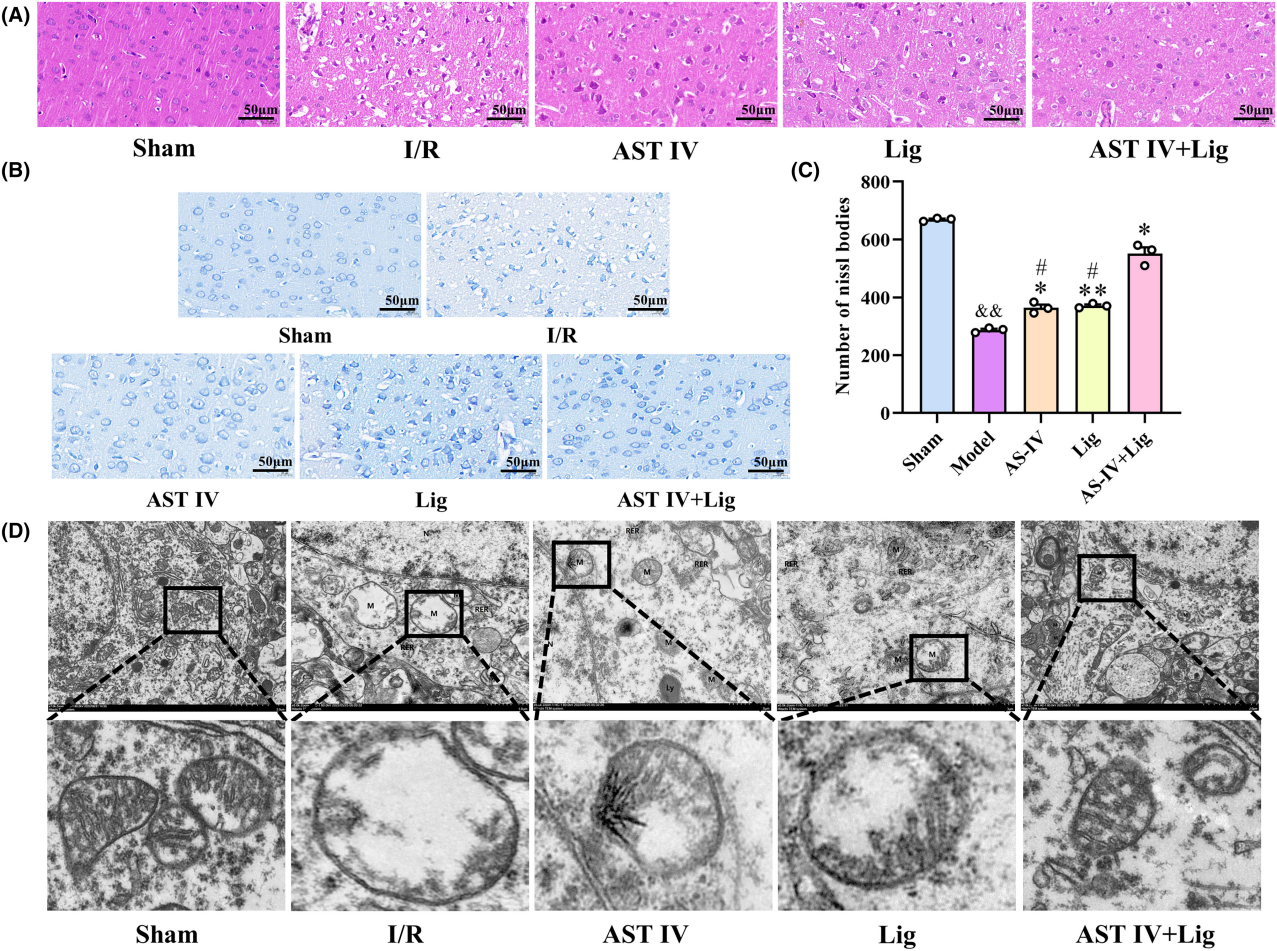
AST IV, Lig, and AST IV + Lig alleviated pathological damages in CIRI rats. (A) The morphology of brain nerve cells was observed by HE staining; (B) The morphology of brain nerve cells was observed by Nissl staining (×400, scale bar: 50 μm); (C) The relative number of Nissl bodies. (D) The morphology of mitochondria was observed by TEM. ^&&^ *p* < 0.01, versus sham group; **p < 0.01, versus I/R group; *p < 0.05, versus I/R group; ^*#*^ *p* < 0.05, versus AST IV + Lig group, *n* = 3.

HE and Nissl staining were used to detect neuronal damage in the ischemic cortex of rats (Figure 3). In the sham group, the cortical cells were neatly arranged, with intact cell morphology, uniform staining, and clear nuclear contours. No obvious nuclear atrophy or damage was observed. Compared with the sham group, the cortical cells in the I/R group were sparsely distributed, forming many empty spaces, and the cell structure was severely damaged, with nuclear deformation, shrinkage, dark staining, and even disappearance. However, compared with the I/R group, the cortical cells' morphology in AST IV, Lig, and AST IV + Lig groups was significantly improved, and vacuolation and nuclear contraction were reduced. Moreover, the improvement effect of the AST IV + Lig group was more obvious than that of AST IV and Lig groups (Figure 3A). Based on Nissl staining, the neurons in the sham group were large in volume, regular in shape, and dense in Nissl bodies. Conversely, Nissl bodies in the I/R group were obviously sparse, irregular in shape, lighter in color, less stained area, more vacuolar degeneration, and fewer (*p* < 0.01). However, compared with the I/R group, the neuron volume in AST IV, Lig, and AST IV + Lig groups became larger, their color became darker, the staining area increased, and the number of Nissl bodies increased significantly (*p* < 0.05, *p* < 0.01, and, *p* < 0.05, respectively). The number of Nissl bodies in the AST IV + Lig group was substantially higher than that in the AST IV and Lig groups (both *p* < 0.05; Figure 3B,C). The mitochondria morphology in the ischemic cortex of rats was observed using TEM (Figure 3D). In the sham group, the mitochondria (M) were moderately abundant, mostly round or short rod‐shaped, and slightly swollen, with intact membranes, and a slight decrease in matrix density. In contrast, mitochondria (M) in the I/R group exhibited severe swelling, increased volume, matrix lysis, cristae disappearance, and vacuolization. However, compared with the I/R group, the mitochondria (M) morphology was improved in AST IV, Lig, and AST IV + Lig groups, with moderate or mild swelling, partial cristae rupture and shrinkage, and local decrease in matrix density. The improvement of mitochondrial morphology in the AST IV + Lig group was more obvious than that in AST IV and Lig groups.

### 3.4 AST IV and Lig downregulated Drp1 SUMO‐1ylation and upregulated Drp1 SUMO‐2/3ylation in CIRI rats

**FIGURE 4:**
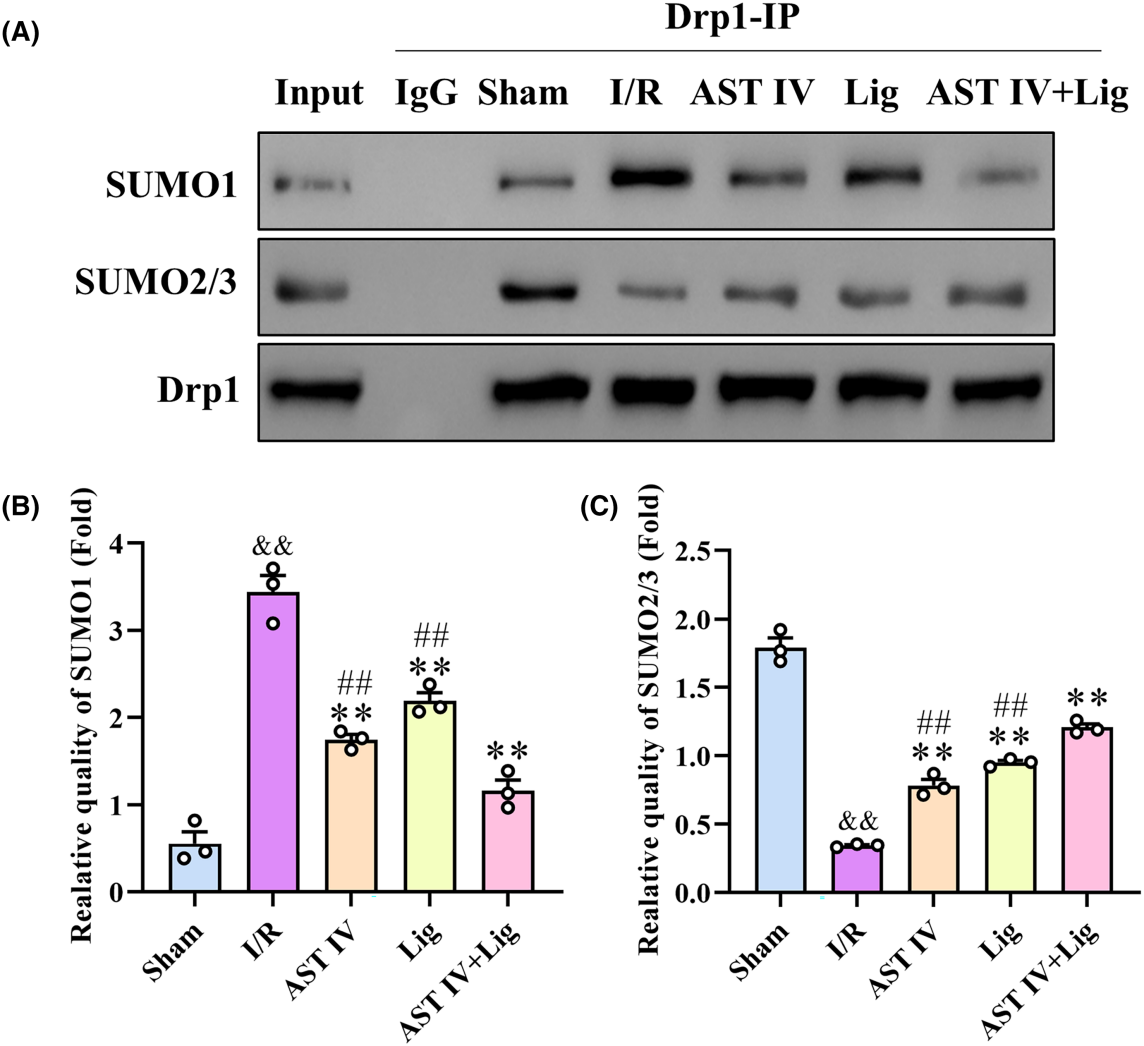
AST IV, Lig, and AST IV + Lig downregulated SUMO‐1ylation of Drp1 and upregulated SUMO‐2/3ylation of Drp1. (A) Protein bands of SUMO1, SUMO2/3 and Drp1; (B) Relative quality of SUMO1; (C) Relative quality of SUMO2/3. ^&&^ *p* < 0.01, versus sham group; **p < 0.01, versus I/R group; ^*##*^ *p* < 0.01, versus AST IV + Lig group, *n* = 3.

The binding levels between Drp1 and SUMO1, as well as Drp1 and SUMO2/3, were examined using Co‐IP. Figure 4 illustrates that compared with the sham group, the binding of Drp1 to SUMO1 was significantly increased (*p* < 0.01), whereas that of Drp1 to SUMO2/3 was significantly decreased in the I/R group (*p* < 0.01). Compared with the I/R group, the binding of Drp1 to SUMO1 was significantly decreased (*p* < 0.01), while that of Drp1 to SUMO2/3 was significantly increased in all treated groups (*p* < 0.01). The binding level between Drp1 and SUMO1 in the AST IV + Lig group was considerably lower than those in AST IV and Lig groups (both *p* < 0.01). However, the binding level between Drp1 and SUMO2/3 in the AST IV + Lig group was significantly higher than those in AST IV and Lig groups (both *p* < 0.01).

### 3.5 AST IV and Lig inhibited mitochondrial fission and promoted mitochondrial fusion in CIRI rats

**FIGURE 5:**
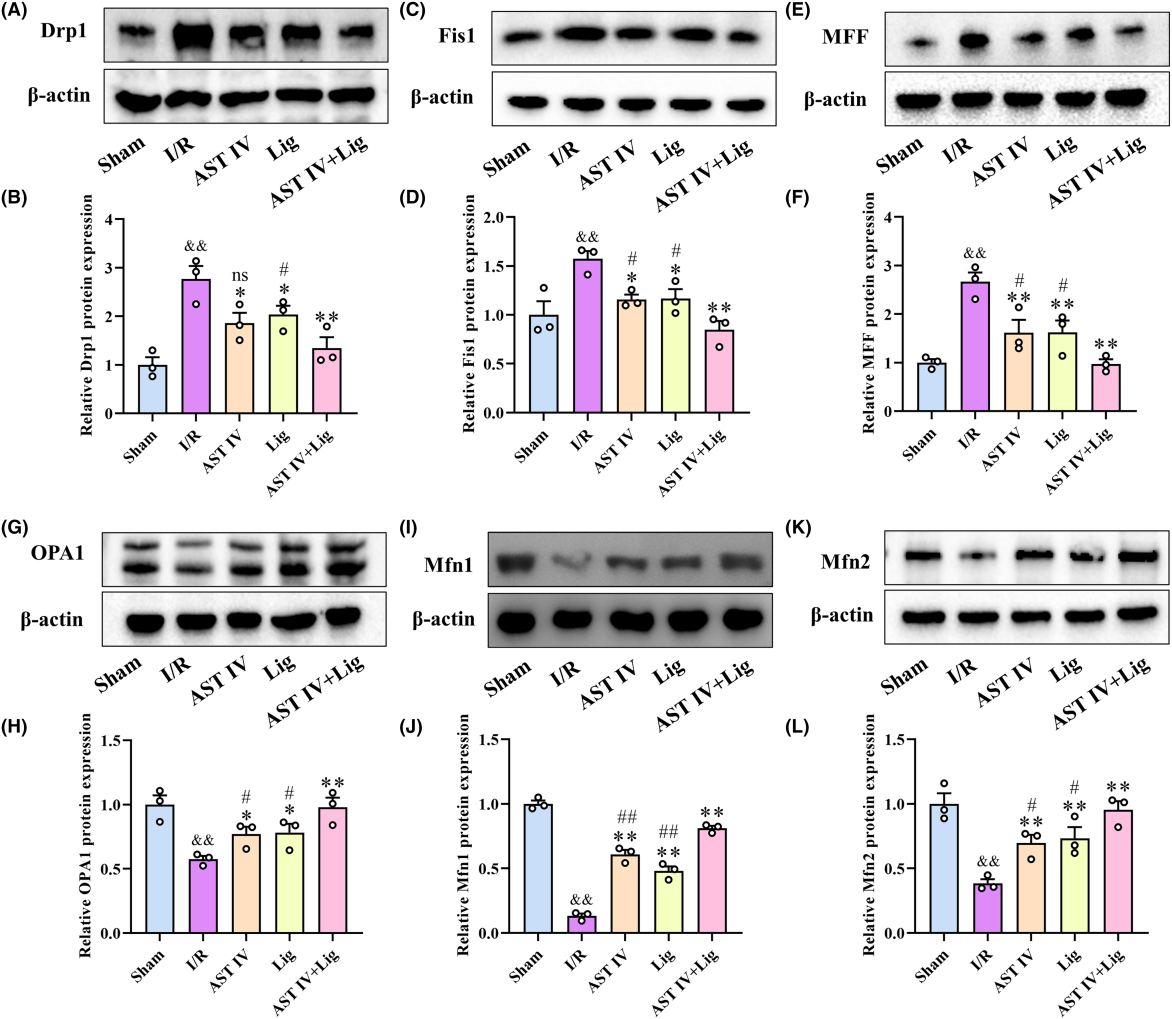
AST IV, Lig, and AST IV + Lig reduced Drp1, Fis1, MFF expressions and enhanced OPA1, Mfn1/2 expressions. (A, B) Protein expression of Drp1 in each group; (C, D) Protein expression of Fis1 in each group; (E, F) Protein expression of MFF in each group; (G, H) Protein expression of OPA1 in each group; (I, J) Protein expression of Mfn1 in each group; (K, L) Protein expression of Mfn2 in each group. ^&&^ *p* < 0.01, versus sham group; **p < 0.01, versus I/R group; *p < 0.05, versus I/R group; ^*##*^ *p* < 0.01, versus AST IV + Lig group; ^*#*^ *p* < 0.05, versus AST IV + Lig group, *n* = 3.

Western blot analysis was applied to quantify the protein expression levels of Drp1, Fis1, MFF, OPA1, Mfn1, and Mfn2 (Figure 5). Compared with the sham group, the protein expressions of Drp1, Fis1, and MFF in the I/R group were significantly increased (*p* < 0.01), whereas the protein expressions of OPA1, Mfn1, and Mfn2 were significantly decreased (*p* < 0.01). Compared with the I/R group, both the AST IV and Lig groups significantly reduced the expressions of Drp1, Fis1, and MFF (*p* < 0.05, *p* < 0.05, and *p* < 0.01, respectively) and significantly upregulated the expressions of OPA1, Mfn1, and Mfn2 (all *p* < 0.01); AST IV + Lig group extremely considerably reduced the expressions of Drp1, Fis1, and MFF (all *p* < 0.01) and extremely significantly upregulated the expressions of OPA1, Mfn1, and Mfn2 (all *p* < 0.01). Compared with the AST IV group and Lig group, not only Drp1 (*p* < 0.01 and *p* < 0.05, respectively) but also the Fis1 and MFF expressions (both *p* < 0.05) were significantly reduced in the AST IV + Lig group. Concurrently, the expressions of OPA1, Mfn1, and Mfn2 were significantly increased (*p* < 0.05, *p* < 0.01, and *p* < 0.05, respectively).

### 3.6 Optimization of drug dose and administration time in SY5Y cells

**FIGURE 6:**
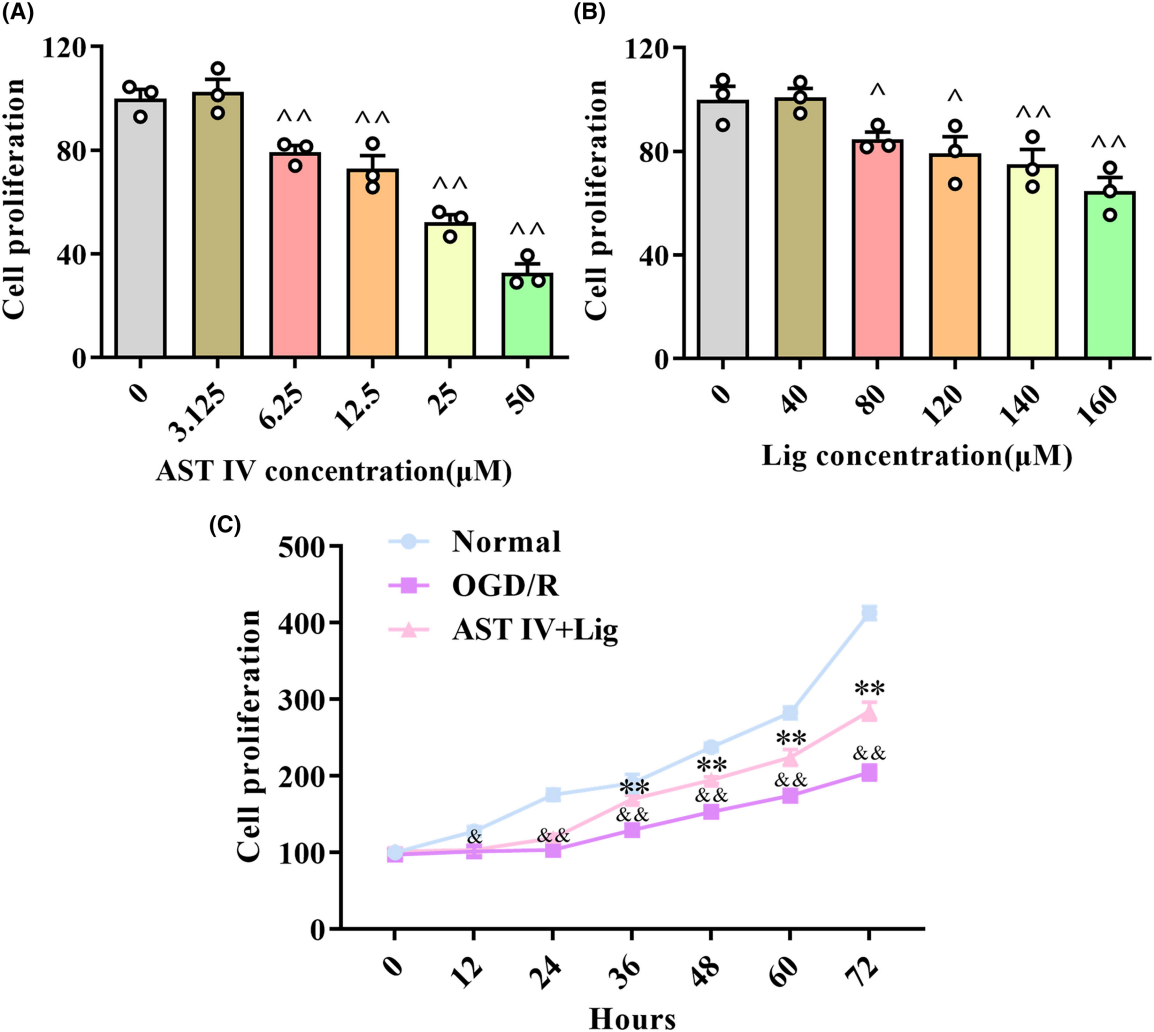
Optimization of drug dose and administration time. (A) Cell proliferation under different concentrations of AST IV and Lig; (B) Cell proliferation under different concentrations of Lig; (C) Cell proliferation under different times of AST IV + Lig. ^*^^*^ *p* < 0.01, versus 0 μM; ^*^*^ *p* < 0.05, versus 0 μM; ^&&^ *p* < 0.01, versus normal group; ^&^ *p* < 0.05, versus normal group; **p < 0.01, versus OGD/R group; *p < 0.05, versus OGD/R group. *n* = 3.

CCK‐8 was used to assess the effects of AST IV and Lig on SH‐SY5Y cell proliferation to select the appropriate concentration and administration time (Figure 6). The results revealed that AST IV at 6.25 μM significantly inhibited cell proliferation (*p* < 0.01) (Figure 6A), while Lig at 80 μM exerted a significant inhibitory effect on cell proliferation (*p* < 0.05) (Figure 6B). Compared with the OGD/R group, the AST IV + Lig group remarkably alleviated the inhibition of cell proliferation after 36 h (*p* < 0.01) (Figure 6C). Thus, 36 h was chosen as the administration time.

### 3.7 AST IV combined with Lig decreased LDH and ROS content, increased ATP content, and mitochondrial membrane potential in SY5Y cells

**FIGURE 7:**
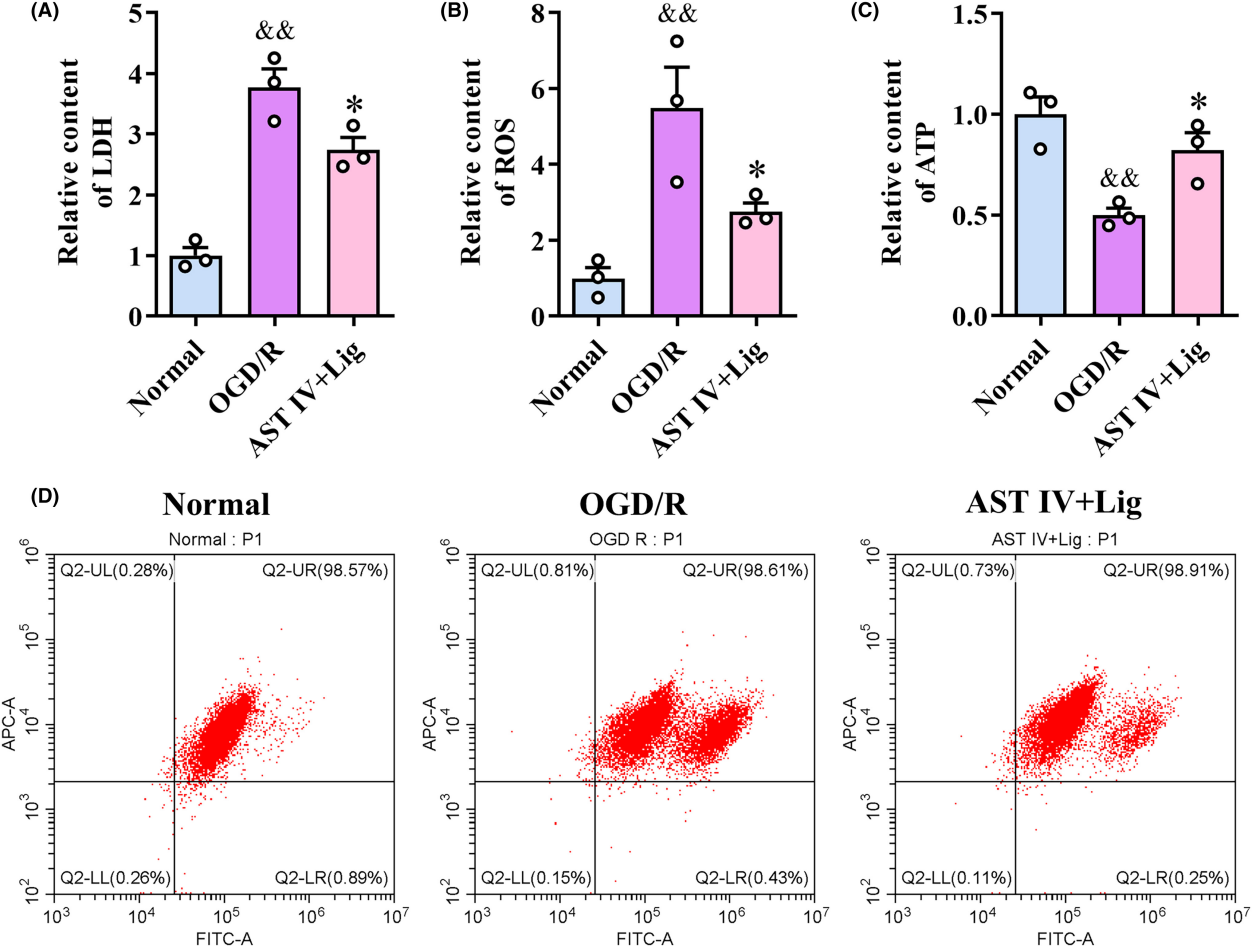
AST IV + Lig alleviated OGD/R‐induced neuronal and mitochondrial damage. (A–C) Observation of LDH, ROS and ATP content in each group; (D) Observation of mitochondrial membrane potential. ^&&^ *p* < 0.01, versus normal group; *p < 0.05, versus OGD/R group. *n* = 3.

LDH, ROS, ATP content, and mitochondrial membrane potential were measured to observe cell and mitochondrial damage (Figure 7). Compared with the normal group, in the OGD/R group, LDH and ROS levels were significantly increased (all *p* < 0.01), ATP levels were significantly decreased (*p* < 0.01), and mitochondrial membrane permeability was considerably increased, indicating a substantial decrease in mitochondrial membrane potential. Compared with the OGD/R group, the AST IV + Lig group exhibited a notable reduction in LDH and ROS levels (*p* < 0.05), a significant increase in ATP levels (*p* < 0.05), and a substantial decrease in mitochondrial membrane permeability, indicating a considerable increase in mitochondrial membrane potential.

### 3.8 AST IV combined with Lig regulated mitochondrial dynamics and SENPs‐related proteins

**FIGURE 8:**
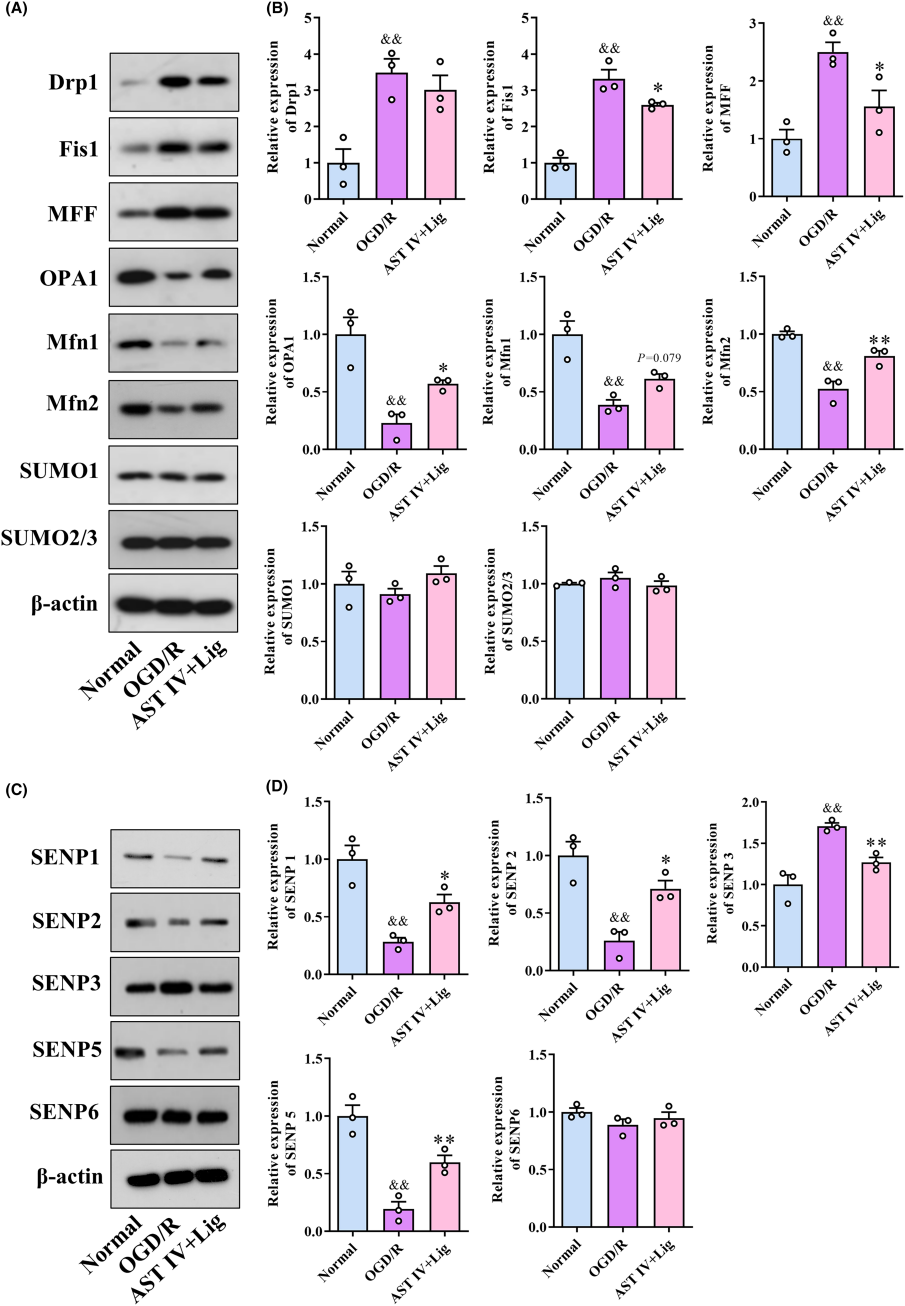
AST IV + Lig regulated mitochondrial dynamics and SENPs‐related proteins. (A, B) Protein expression of Drp1, Fis1, MFF, OPA1, Mfn1, Mfn2, SUMO1, and SUMO2/3 in each group; (C, D) Protein expression of SENP1, SENP2, SENP3, SENP5, and SENP6 in each group. ^&&^ *p* < 0.01, versus normal group; **p < 0.01, versus OGD/R group; *p < 0.05, versus OGD/R group. *n* = 3.

Western blot analysis was applied to quantify protein expressions (Figure 8). Compared to the normal group, the expressions of Drp1, Fis1, MFF, and SENP3 were significantly increased (all *p* < 0.01) in the OGD/R group, whereas those of OPA1, Mfn1, Mfn2, SENP1, SENP2, and SENP5 were significantly decreased (all *p* < 0.01). Compared to the OGD/R group, the AST IV + Lig group showed a significant decrease in the expressions of Fis1, MFF, and SENP3 (*p* < 0.05, *p* < 0.05 and *p* < 0.01, respectively) and a significant increase in those of OPA1, Mfn2, SENP1, SENP2, and SENP5 (*p* < 0.05, *p* < 0.01, *p* < 0.05, *p* < 0.05, and *p* < 0.01, respectively). No statistically significant differences were observed in the expressions of SUMO1, SUMO2/3, and SENP6 among the three groups.

### 3.9 AST IV combined with Lig regulated Drp1 SUMOylation, correcting mitochondrial dynamics

**FIGURE 9:**
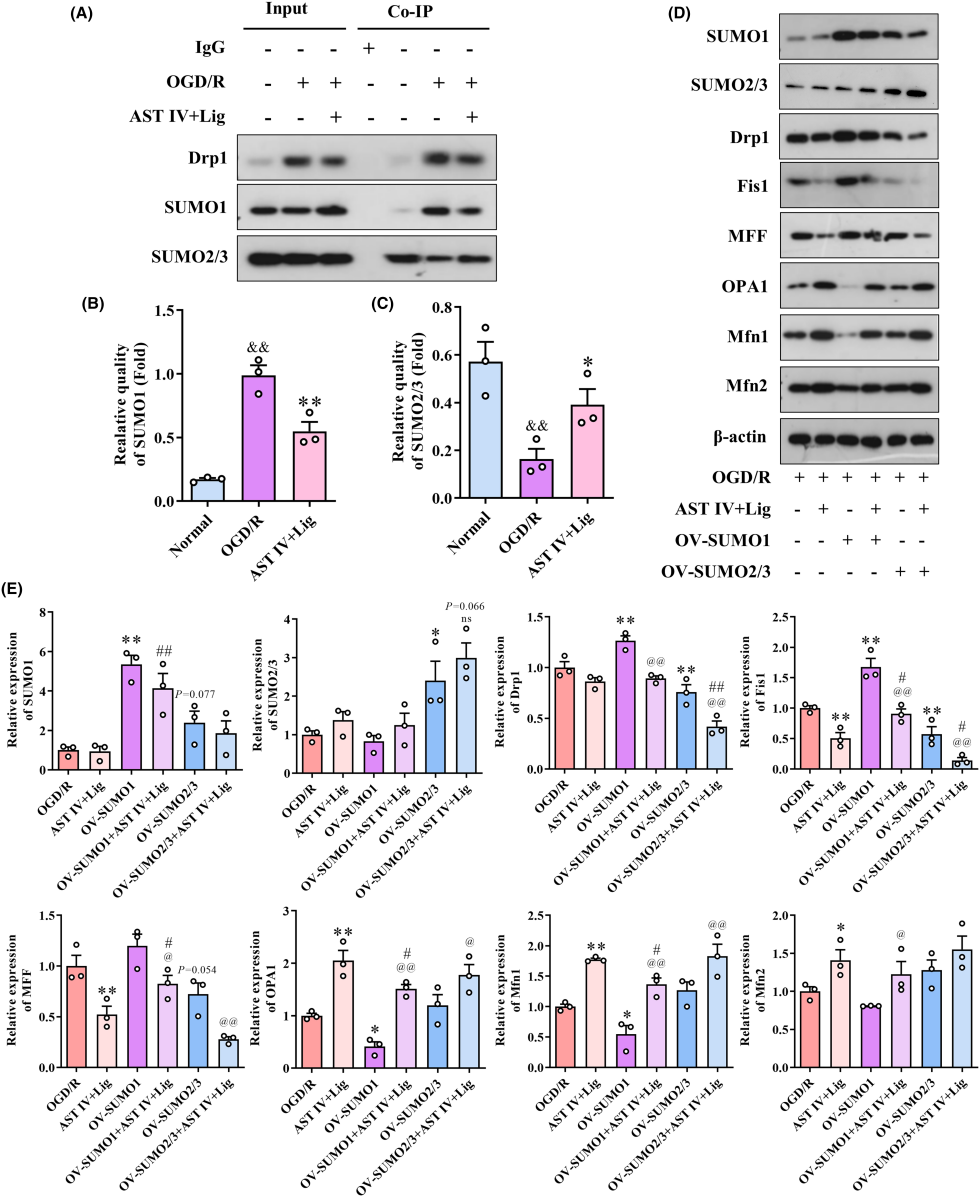
AST IV + Lig regulates Drp1 SUMOylation, correcting mitochondrial dynamics. (A–C) Co‐IP was used to detect the SUMOylation of Drp1; (D, E) Western blot was used to detect the expression of SUMO1, SUMO2/3, Drp1, Fis1, MFF, OPA1, Mfn1, and Mfn2. **p < 0.01, versus OGD/R group; *p < 0.05, versus OGD/R group; ^*##*^ *p* < 0.01, versus AST IV + Lig group; ^*#*^ *p* < 0.05, versus AST IV + Lig group; ^*@@*^ *p* < 0.01, versus Self OV‐SUMO group; ^*@*^ *p* < 0.05, versus Self OV‐SUMO group. *n* = 3.

Drp1 SUMOylation was examined using Co‐IP (Figure 9A–C). Compared with the normal group, the SUMO1 modification of Drp1 was significantly increased in the OGD/R group (*p* < 0.01), whereas the SUMO2/3 modification of Drp1 was significantly decreased (*p* < 0.01). However, compared with the OGD/R group, the SUMO1 modification of Drp1 was reduced considerably in the AST IV + Lig group (*p* < 0.01), whereas the SUMO2/3 modification of Drp1 was significantly increased (*p* < 0.05). SUMO1 and SUMO2/3 overexpressions were used to observe the effects of SUMO protein on mitochondrial dynamics (Figure 9D,E). Compared with the OGD/R group, Fis1 and MFF expressions were significantly reduced in the AST IV + Lig group (both *p* < 0.01), and those of OPA1, Mfn1, and Mfn2 were significantly decreased (*p* < 0.01, *p* < 0.01, and *p* < 0.05, respectively). In the OV‐SUMO1 group, the expressions of SUMO1, Drp1, and Fis1 were significantly increased (all *p* < 0.01), whereas those of OPA1 and Mfn1 were decreased considerably (both *p* < 0.05). In the OV‐SUMO2/3 group, the expression of SUMO2/3 was significantly increased (*p* < 0.05), whereas the protein expressions of Drp1 and Fis1 were decreased considerably (both *p* < 0.01). Compared with the AST IV + Lig group, in the OV‐SUMO1 + AST IV + Lig, the expressions of SUMO1, Fis1, and MFF group were significantly increased (*p* < 0.01, *p* < 0.05, and *p* < 0.05, respectively), whereas those of OPA1 and Mfn1 were decreased considerably (both *p* < 0.05). In the OV‐SUMO2/3 + AST IV + Lig group, the expressions of Drp1 and Fis1 were significantly reduced (*p* < 0.01 and *p* < 0.05, respectively). Compared with the OV‐SUMO1 group, in the OV‐SUMO1 + AST IV + Lig group, the expressions of Drp1, Fis1, and MFF were significantly decreased (*p* < 0.01, *p* < 0.01, and *p* < 0.05, respectively), whereas those of OPA1, Mfn1, and Mfn2 were significantly increased (*p* < 0.01, *p* < 0.01, and *p* < 0.05, respectively). Compared with the OV‐SUMO2/3 group, the expressions of Drp1, Fis1, and MFF were significantly decreased (all *p* < 0.01), whereas those of OPA1 and Mfn1 were significantly increased (*p* < 0.05 and *p* < 0.01, respectively) in the OV‐SUMO2/3 + AST IV + Lig group.

## 4 DISCUSSION

Reperfusion after ischemic stroke triggers oxidative stress, mitochondrial dysfunction, and neuronal programmed death, resulting in CIRI. Subsequently, CIRI initiates a series of pathological responses, exacerbating neuronal damage. Therefore, mitigating CIRI is crucial when treating ischemic stroke. AST IV and Lig play a beneficial role in preventing and treating CIRI.^40^, ^41^, ^42^ Moreover, AST IV combined with Lig has a good synergistic effect; however, the underlying mechanisms remain unknown. Therefore, the rat MCAO/R model and the SY5Y cell OGD/R model were established to investigate the effects and potential mechanisms of AST IV combined with Lig on CIRI. Consequently, AST IV, Lig, and AST IV combined with Lig were found to improve neural and cognitive functions, increase CBF recovery, reduce the volume of cerebral infarction, and alleviate neuronal and mitochondrial damage in CIRI rats. Concurrently, AST IV combined with Lig exhibited a better effect than the single drug, indicating a good synergistic effect, as per previous research.^32^ Not only could the AST IV and Lig combination enhance the cell vitality, reduce LDH and ROS levels, increase ATP levels, and elevate mitochondrial membrane potential in SH‐SY5Y cells, but it also could reduce the binding of Drp1 to SUMO1, increase the binding of Drp1 to SUMO2/3, inhibit the expressions of Drp1, Fis1, MFF, and SENP3, and increase the expressions of OPA1, Mfn1, Mfn2, SENP1, SENP2, and SENP5. Therefore, AST IV combined with Lig could reduce Drp1 SUMO‐1ylation, increase Drp1 SUMO‐2/3ylation, inhibit mitochondrial fission, promote mitochondrial fusion, and ameliorate abnormal mitochondrial dynamics, thus alleviating CIRI. SENP1, SENP2, and SENP5 were involved in the Drp1 deSUMO‐1ylation, and SENP3 contributed to the Drp1 deSUMO‐2/3ylation.

AST IV combined with Lig could improve neural and cognitive functions, reduce the volume of cerebral infarction, and alleviate neuronal damage in CIRI rats. CIRI is a complex pathological process involving various mechanisms, including inflammation, excitotoxicity of amino acids, calcium overload, energy metabolism disorders, and oxidative stress, directly or indirectly leading to cerebral infarction and the loss of neurological and cognitive function.^43^ Moreover, 24 h after cerebral I/R in rats, CBF in the cerebral cortex was found to be negatively correlated with the severity of brain tissue damage in a prior study.^44^ This was consistent with the outcomes of our research. Meanwhile, AST IV, Lig, and AST IV combined with Lig were demonstrated to increase CBF and reduce brain infarct volume and tissue damage. Furthermore, AST IV combined with Lig enhanced cell viability and decreased LDH levels. These findings indicated that AST IV combined with Lig exhibited a favorable neuroprotective effect in CIRI.

AST IV combined with Lig could enhance mitochondrial structure and function. Mitochondria, as highly dynamic intracellular organelles, play a crucial role in mediating the pathophysiology of ischemic neuronal death.^45^ Mitochondrial morphology is closely associated with mitochondrial function. CIRI causes mitochondria fragmentation,^46^ possibly resulting in energetic disruption, oxidative stress, and ROS overproduction. Moreover, excessive ROS can induce the opening of mitochondrial transition pores (mPTP), resulting in impaired mitochondrial membrane potential and ultimately aggravating tissue injury.^47^ Prior studies found that AST IV^48^, ^49^ and Lig^30^, ^31^ could alleviate I/R‐induced apoptosis by maintaining the mitochondrial structural and functional integrity. The current study demonstrated that AST IV combined with Lig significantly reversed the increase in ROS and decrease in ATP and mitochondrial membrane potential induced by OGD/R. Moreover, AST IV, Lig, and AST IV combined with Lig reversed mitochondrial swelling, matrix lysis, and cristae disruption in rat CIRI cortical neurons. Obviously, the effects of AST IV combined with Lig were better than those of the single drug. These results indicated that AST IV combined with Lig could improve mitochondrial structure and function, thereby maintaining mitochondrial homeostasis.

AST IV combined with Lig could correct the mitochondrial dynamics imbalance. Prior research proved that regulating MQC, especially mitochondrial dynamics, is significantly important in alleviating the pathophysiological injury of damaged mitochondria on neurons.^50^ Mitochondrial dynamics play an indispensable role in determining cell fate by maintaining mitochondrial homeostasis. Mitochondrial fission maintains the stability of the number and proper distribution of mitochondria in cells. In contrast, mitochondrial fusion ensures optimal mitochondrial activity by allowing the exchange of contents between fusing mitochondria.^51^ An imbalance in mitochondrial dynamics results in an alteration of mitochondrial quantity, morphology, and function, leading to CIRI. Drp1, a key protein involved in mitochondrial fission, is translocated to the surface of the mitochondrial outer membrane and interacts with Fis1 and MFF upon activation of mitochondrial fission.^52^ Meanwhile, more Drp1s are recruited to achieve mitochondrial inner and outer membrane fission.^53^ Mfn1/2, located in the mitochondrial outer membrane, and OPA1, located in the mitochondrial inner membrane, are involved in mediating mitochondrial fusion.^54^ Mfn1/2 promotes the docking of mitochondrion, and OPA1 maintains the morphology of mitochondrial cristae.^55^, ^56^ Our results, both in animal and cell experiments, revealed that CIRI increased the expressions of Drp1, Fis1, and MFF while decreasing the expressions of OPA1, Mfn1, and Mfn2. Conversely, AST IV, Lig, and AST IV combined with Lig reversed this situation. The efficacy of AST IV combined with Lig was visibly better than that of the single drug, exhibiting a good synergistic effect. Consequently, the results indicated that CIRI induced neuronal mitochondrial fission and reduced mitochondrial fusion. AST IV combined with Lig could correct the mitochondrial dynamics imbalance by inhibiting mitochondrial fission and promoting mitochondrial fusion, exerting a protective effect against CIRI.

AST IV combined with Lig could reduce Drp1 SUMO‐1ylation and increase Drp1 SUMO‐2/3ylation in CIRI. During CIRI, cellular protein properties and functions can be dynamically reprogrammed through posttranslational protein modifications in response to homeostatic imbalance. SUMOylation, one of the posttranslational modifications, can regulate protein activity, stability, localization, interaction, and function.^57^ By mediating SENPs, SUMO can be dissociated from the substrate protein. There are five SUMOs in mammals, namely, SUMO1–SUMO5.^58^ SUMO1–SUMO3 are widely expressed in brain tissues, while SUMO2 and SUMO3 are commonly referred to as SUMO2/3, sharing 96% homology.^59^ SENP1 and SENP2 can dissociate SUMO1 and SUMO2/3 from target proteins. The SENP family comprises SENP1‐3 and SENP5‐8. Various SENPs have dissociative effects on different SUMOs that bind to substrate proteins. The dynamic balance between SUMOylation and deSUMOylation is critical for maintaining the normal physiological functions of substrate proteins. The imbalance will lead to abnormal function of substrate proteins, contributing to disease occurrence.^60^ Longitudinal studies displayed that SUMOylation can interfere with mitochondrial dynamics, a crucial process for normal neuronal function.^61^ SUMO1 overexpression and the knockout of SENP1 can increase Drp1‐SUMO1 coupling and promote the mitochondrial localization of Drp1, resulting in mitochondrial fragmentation and increasing mitochondrial pathway apoptosis.^16^ SENP2 and SENP5 mediate the Drp1 deSUMOylation, thereby reducing the stability of Drp1, decreasing mitochondrial fragmentation, and maintaining mitochondrial structure and function.^20^ Mitochondrial fission can be enhanced by knocking out SENP2 by promoting SUMO‐1ylation of Drp1.^21^ SENP3 plays a role in deSUMO‐2/3ylation by reducing Drp1‐SUMO2/3 coupling, which promotes the mitochondrial localization of Drp1, resulting in mitochondrial fragmentation, the release of cytochrome C, initiation of apoptosis,^17^ and the release of LDH, triggering cell death.^18^ Knockout SENP3 increases Drp1‐SUMO2/3 coupling, which reduces caspase‐3 and LDH. SENP3 overexpression promotes Drp1 mitochondrial localization and increases the release of cytochrome C.^19^ In this study, the results of Co‐IP revealed that CIRI induced an increase in the binding of Drp1 to SUMO1 and a decrease in the binding of Drp1 to SUMO2/3. During the CIRI, the reduction of SENP1, SENP2, and SENP5 and the increase of SENP3 indicated that deSUMO‐1ylation might be regulated by SENP1, SENP2, and SENP5 and deSUMO‐2/3ylation might be regulated by SENP3. These results were consistent with those of previous studies.^62^, ^63^ Fortunately, AST IV, Lig, and AST IV combined with Lig reversed the changes in SUMOylation of Drp1 induced by CIRI, while the AST IV combined with Lig corrected the alterations in SENPs. Furthermore, OV‐SUMO1 was found to promote the expression of mitochondrial fission proteins and inhibit the expression of mitochondrial fusion proteins, while OV‐SUMO2/3 suppressed the expression of mitochondrial fission proteins. However, AST IV combined with Lig could reverse OV‐SUMO1‐induced mitochondrial fission and enhance the inhibition of mitochondrial fission by OV‐SUMO2/3, promoting mitochondrial fusion. The above findings indicated that the protective effects of AST IV combined with Lig on CIRI might be achieved by reducing SUMO‐1ylation of Drp1 and increasing SUMO‐2/3ylation of Drp1 to inhibit mitochondrial fission and promote mitochondrial fusion.

Studies have shown that oxidative stress caused by CIRI can lead to mitochondrial dysfunction, specifically impairing mitochondrial oxidative phosphorylation. This dysfunction is further exacerbated by the production and buildup of ROS, which disrupt the electrochemical gradient across mitochondrial membranes, resulting in decreased ATP production, reduced mitochondrial membrane potential, mitochondrial swelling, and ultimately, structural and functional damage to the mitochondria.^64^ Additionally, research has demonstrated a significant upregulation of SENP3 expression in NRK‐52E cells following lipopolysaccharide (LPS) treatment, in response to the accumulation of ROS. SENP3 facilitates Drp1 deSUMO‐2/3ylation in order to enhance mitochondrial fission within renal tubular epithelial cells, thereby exacerbating apoptosis of cells. Depletion of SENP3 results in a reversal of the increase in cleaved caspase 3 and cytochrome C, as well as the decrease in mitochondrial membrane potential in LPS‐induced NRK‐52E cells.^62^ In mice with myocardial ischemia–reperfusion injury, upregulation of SENP3 expression in the heart is contingent upon ROS generation, consequently promoting Drp1 mitochondrial translocation.^65^ Hence, it is hypothesized that the release of ROS in CIRI could play a significant role in the upregulation of SENP3 and subsequent reduction of Drp1 SUMO‐2/3ylation, ultimately exacerbating mitochondrial structural and functional impairment. Alterations in ATP production and mitochondrial membrane potential may also play a pivotal role in the regulation of Drp1 SUMO/deSUMOylation, which remains to be further investigated in our future research.

## 5 CONCLUSION

**FIGURE 10:**
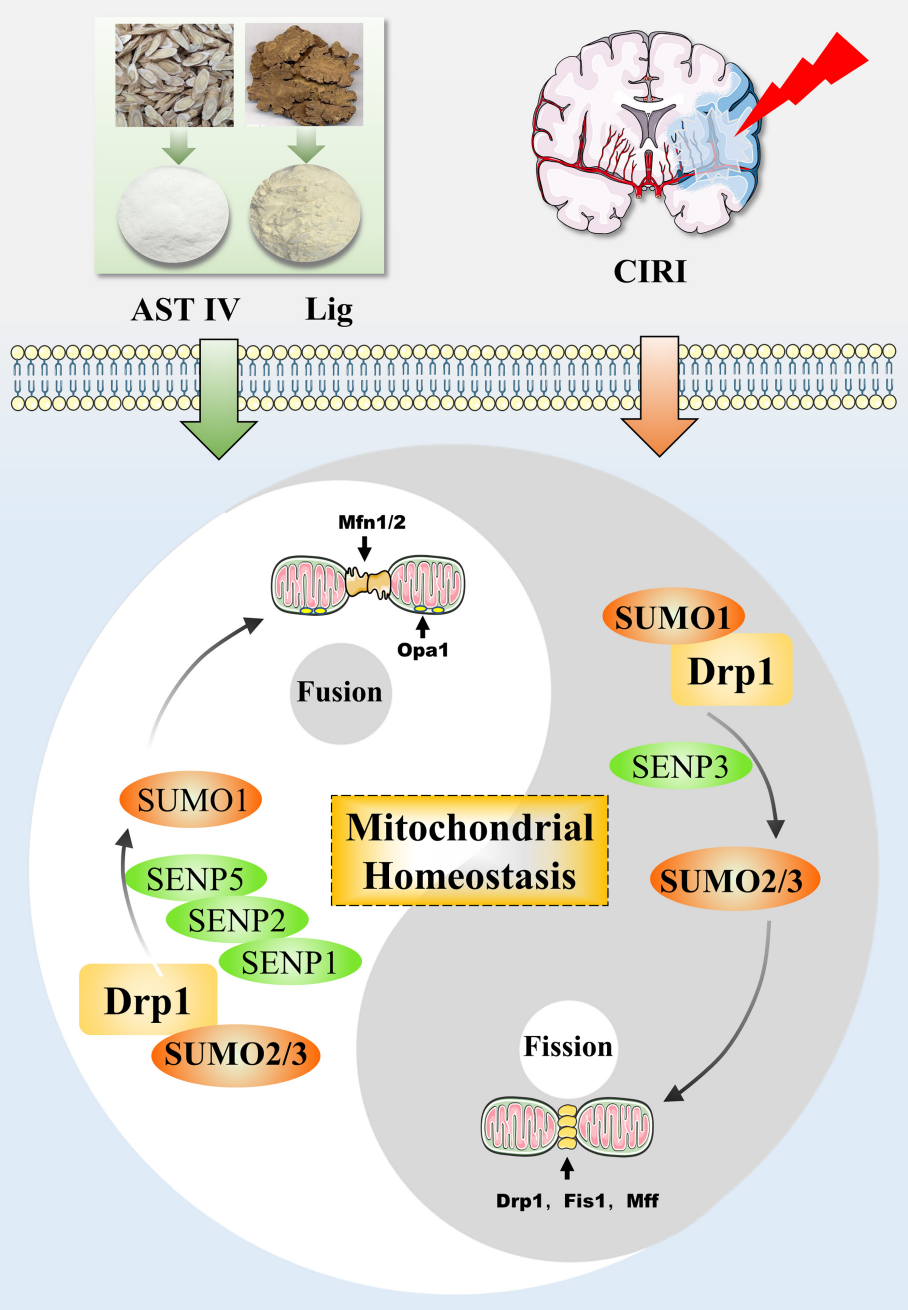
Possible mechanism of action of AST IV combined with Lig in CIRI.

The protective effects and underlying mechanism of AST IV combined with Lig against CIRI by Drp1 SUMO/deSUMOylation and mitochondrial dynamics were explored. Our results revealed that AST IV combined with Lig could ameliorate abnormal mitochondrial dynamics by reducing Drp1 SUMO‐1ylation and increasing Drp1 SUMO‐2/3ylation in CIRI (Figure 10). Regulating Drp1 SUMOylation to mitochondrial dynamics may provide a new treatment strategy for the prevention and treatment of CIRI.

## AUTHOR CONTRIBUTIONS

Wenli Zhang and Zhigang Mei conceptualized the study; Xiangyu Chen, Tong Yang, and Yue Zhou performed investigative studies and data analysis; Xiangyu Chen wrote the manuscript; Zhigang Mei and Wenli Zhang revised the manuscript. All authors read and approved the final manuscript.

## FUNDING INFORMATION

This work was supported by the National Natural Science Foundation of China (82174167), the Project of Natural Science Foundation of Hunan Province (2023JJ30464), the Young Qihuang Scholar Support Project of National Administration of Traditional Chinese Medicine (2022), and the fund for Youth Top Talent Project of Hubei Provincial Health and Family Planning Commission (EWT‐2019‐48).

## CONFLICT OF INTEREST STATEMENT

The authors declare that they have no conflicts of interest.

## Supporting information


Appendix S1

## Data Availability

Data relevant to support the current study can be obtained from our corresponding authors.
