# Supplementary material for: Astragaloside IV combined with ligustrazine ameliorates abnormal mitochondrial dynamics via Drp1 SUMO/deSUMOylation in cerebral ischemia–reperfusion injury
Source: CNS Neurosci Ther. 2024 Apr 14;30(4):e14725. doi: 10.1111/cns.14725 (PMC11016344; doi:10.1111/cns.14725)
Supplement: Supplementary file 1 — Appendix S1 [file CNS-30-e14725-s001.pdf]

Manuscript ID CNSNT-2024-156

Astragaloside IV combined with *ligustrazine* ameliorates abnormal mitochondrial dynamics via Drp1 SUMO/deSUMOylation in cerebral ischemia-reperfusion injury

Xiangyu Chen[1,2], Tong Yang[1], Yue Zhou[1, 3], Zhigang Mei[1, 4, *], Wenli Zhang[5, *]

Full unedited gel/blot for Figure 4A.
The cropped image in the manuscript were highlighted in red

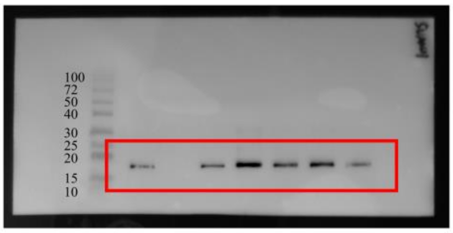

SUMO1
(17kDa)

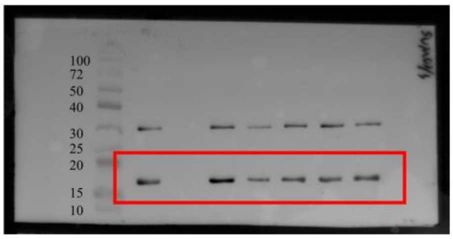

SUMO2/3
(17kDa)

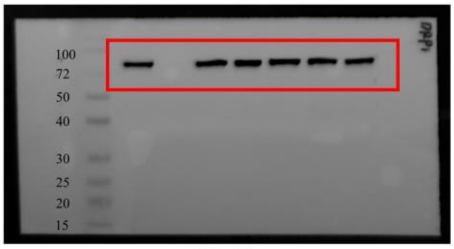

Drp1
(80kDa)

Full unedited gel/blot for Figure 5

The cropped image in the manuscript were highlighted in red

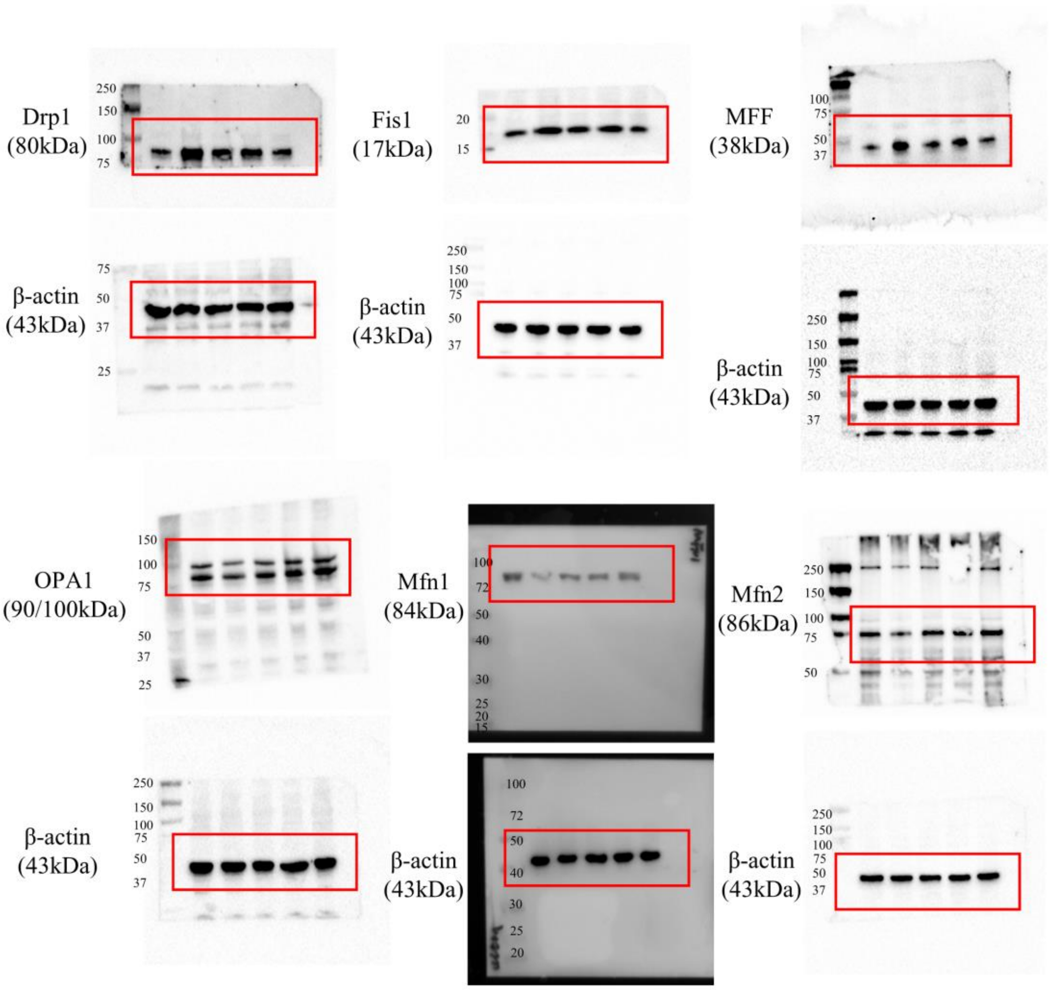

Full unedited gel/blot for Figure 8A.

The cropped image in the manuscript were highlighted in red

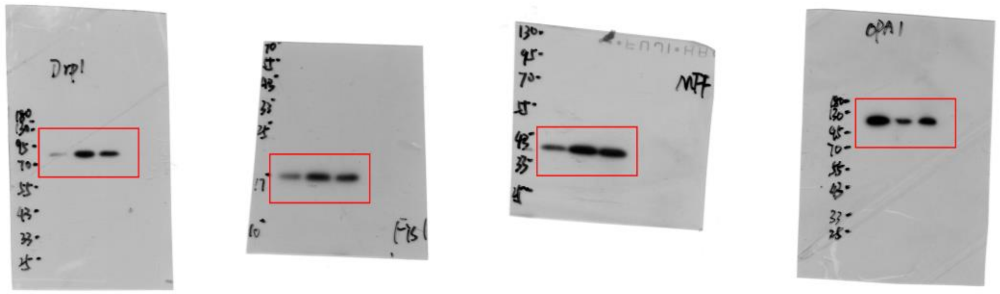

Drp1 (80kDa) | Fis1 (17kDa) | MFF (38kDa) | OPA1 (100kDa)

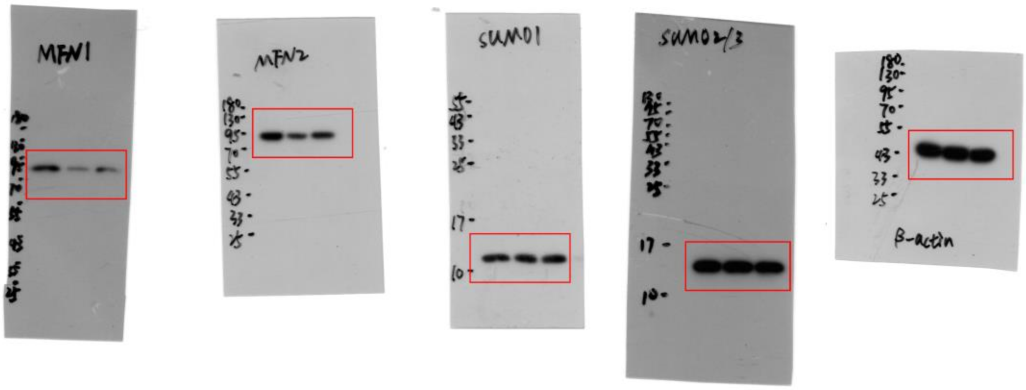

Mfn1 (84kDa) | Mfn2 (86kDa) | SUMO1 (12kDa) | SUMO2/3 (15kDa) | β-actin (43kDa)

Full unedited gel/blot for Figure 8C.

The cropped image in the manuscript were highlighted in red

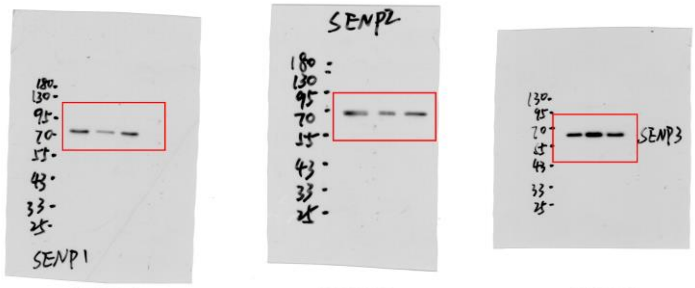

SENP1 (73kDa) | SENP2 (68kDa) | SENP3 (65kDa)

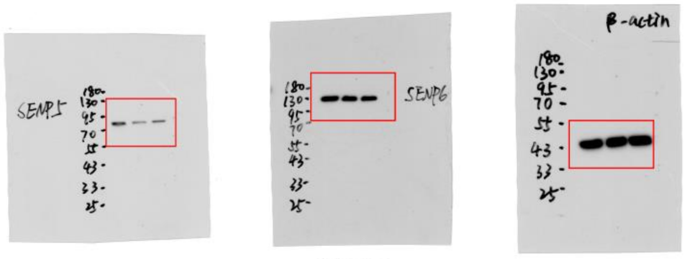

SENP5 (87kDa) | SENP6 (126kDa) | β-actin (43kDa)

Full unedited gel/blot for Figure 9A.
The cropped image in the manuscript were highlighted in red

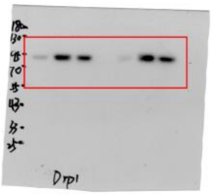
Drp1
(80kDa)

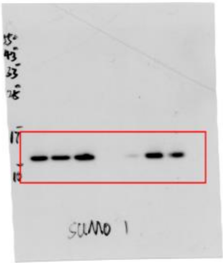
SUMO1
(12kDa)

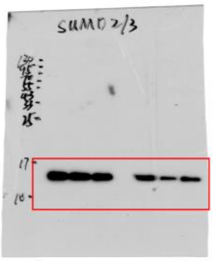
SUMO2/3
(15kDa)

Full unedited gel/blot for Figure 9D.
The cropped image in the manuscript were highlighted in red

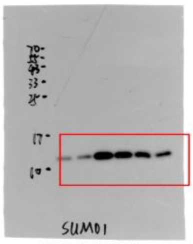
SUMO1
(12kDa)

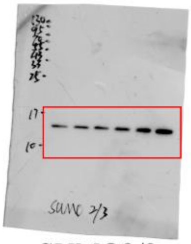
SUMO2/3
(15kDa)

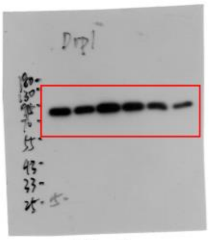
Drp1
(80kDa)

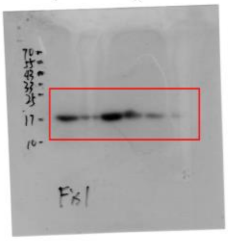
Fis1
(17kDa)

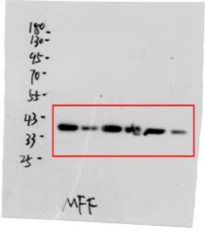
MFF
(38kDa)

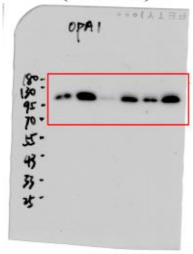
OPA1
(100kDa)

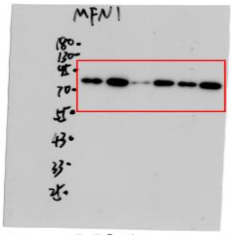
Mfn1
(84kDa)

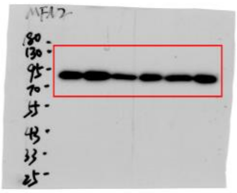
Mfn2
(86kDa)

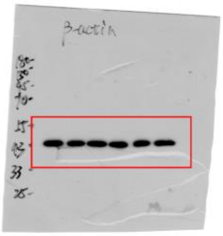
β-actin
(43kDa)
